# Metabolomic studies of *Pseudomonas aeruginosa*

**DOI:** 10.1007/s11274-019-2739-1

**Published:** 2019-11-07

**Authors:** Karolina Anna Mielko, Sławomir Jan Jabłoński, Justyna Milczewska, Dorota Sands, Marcin Łukaszewicz, Piotr Młynarz

**Affiliations:** 1 Bioorganic Chemistry Group, Faculty of Chemistry, Wroclaw University of Science and Technology, Norwida 4/6, 50-373 Wroclaw, Poland; 2 Biotransformation Department, University of Wroclaw, Plac Uniwersytecki 1, 50-137 Wroclaw, Poland; 3 Mother and Child Institute, Kasprzaka 17a, 01-211 Warszawa, Poland

**Keywords:** Metabolomics, Metabolomic experiment, Cystic fibrosis, *Pseudomonas aeruginosa*, Strain identification

## Abstract

**Abstract:**

*Pseudomonas aeruginosa* is a common, Gram-negative environmental organism. It can be a significant pathogenic factor of severe infections in humans, especially in cystic fibrosis patients. Due to its natural resistance to antibiotics and the ability to form biofilms, infection with this pathogen can cause severe therapeutic problems. In recent years, metabolomic studies of *P. aeruginosa* have been performed. Therefore, in this review, we discussed recent achievements in the use of metabolomics methods in bacterial identification, differentiation, the interconnection between genome and metabolome, the influence of external factors on the bacterial metabolome and identification of new metabolites produced by *P. aeruginosa*. All of these studies may provide valuable information about metabolic pathways leading to an understanding of the adaptations of bacterial strains to a host environment, which can lead to new drug development and/or elaboration of new treatment and diagnostics strategies for *Pseudomonas*.

**Graphic abstract:**

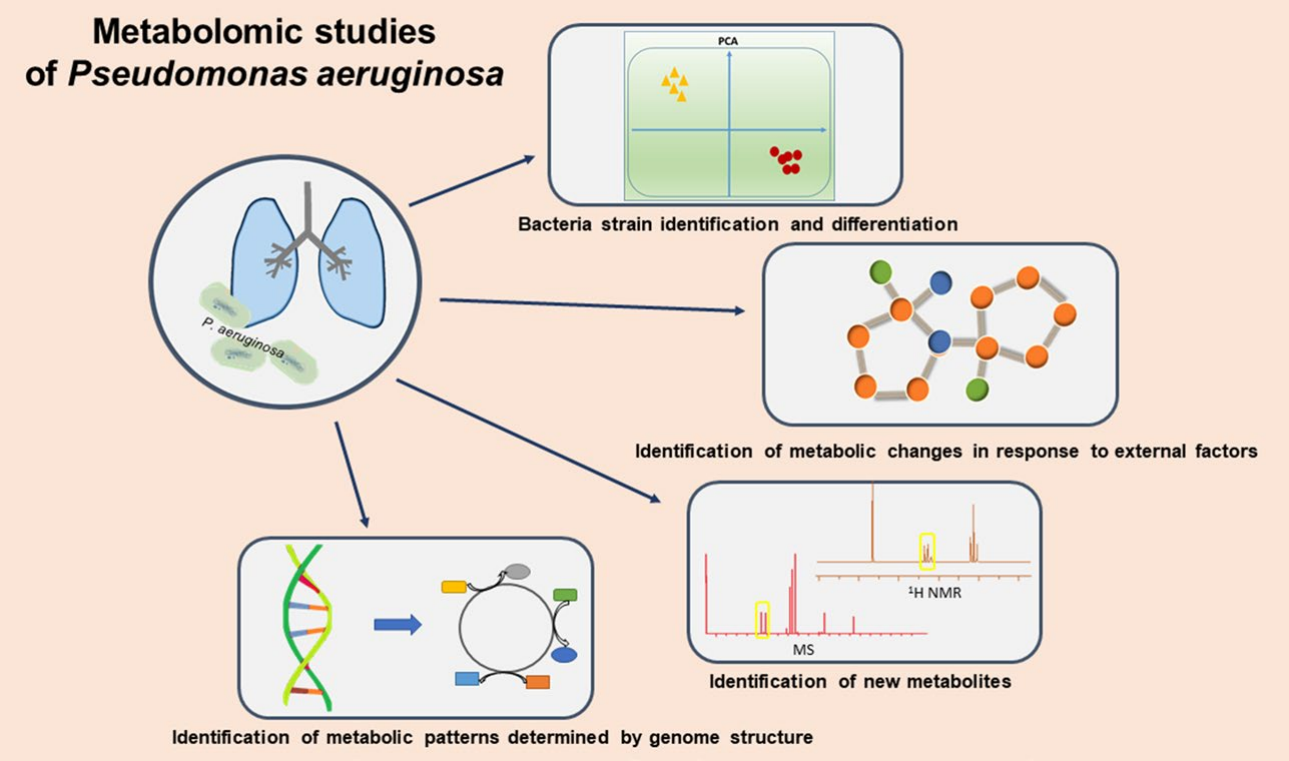

## Introduction

*Pseudomonas aeruginosa* is a common, Gram negative environmental organism. It is often isolated from plants, fruits, soil, and water environments, such as rivers, lakes, and swimming pools. In particular circumstances, *P. aeruginosa* may be a significant pathogenic factor of severe and often opportunistic infections in humans. It typically infects airways and urinary tracts, causes blood infections, and is the most common cause of burn injury infections, hot-tub dermatitis, and outer ear infections (known as swimmer’s ear). *P. aeruginosa* is the most frequent colonizer of medical devices (catheters, nebulizers, humidifiers) and is one of the pathogens that cause nosocomial infections, such as ventilator-associated pneumonia, meningoencephalitis, and sepsis (Bassetti et al. 2018). Treatment of *P. aeruginosa* infections can be difficult due to its natural and acquired resistance to antibiotics (Breidenstein et al. 2011).

*Pseudomonas aeruginosa* is one of the most common organisms isolated from the respiratory tract of cystic fibrosis patients (Bendiak and Ratjen 2009). The occurrence of the infection increases with age and can reach 80% in adults (Behrends et al. 2013). Several studies have shown that this infection leads to higher rates of pulmonary exacerbation and hospitalization in addition to more rapid disease progression, which leads to irreversible and destructive changes in the respiratory system and as a consequence, to chronic respiratory failure. It is also associated with more frequent cystic fibrosis complications, such as malnutrition or diabetes (Emerson et al. 2002; Kosorok et al. 2003; Nixon et al. 2001).

A characteristic feature of the genus *Pseudomonas* is biofilm formation and fluorescent dyes and siderophore production (Leon 1979; Peix et al. 2018; Winstanley et al. 2016). Moreover, microorganisms belonging to this genus show a high capability of utilizing different substrates and a high tendency toward antibiotic resistance. *P. aeruginosa* shows significant adaptation capabilities, as in the case of the development of chronic infections in patients with cystic fibrosis (CF). At this stage, the pathogen is practically impossible to eradicate.

Research on the system biology of *P. aeruginosa* has been carried out for a long time at different levels of molecular organization (genome, transcriptome, and proteome), resulting in detailed information about the genomic structure. The size of the *P. aeruginosa* genome is around 6.5 Mbp. However, the size range for different strains is between 5.2 and 7 Mbp (Schmidt et al. 1996). There are 5021 genes with more than 70% sequence identity between different *P. aeruginosa* strains, and among them, around 4500 genes with > 98% identity. It is suggested that about 4000 genes are common to the majority of the *P. aeruginosa* strains (they are so-called ‘core genome’) (Parkins et al. 2018). The core genome is accompanied by genes that are present in a smaller number of strains. It is estimated that the complete set of genes found in different *P. aeruginosa* strains include between 10,000 and 40,000 genes. The arrangement of the genome may differ between strains; therefore, the identification of regions suitable for gene markers is difficult.

Information about *P. aeruginosa* gene and protein data is available from several databases: (1) the *Pseudomonas* Genome Database, which now has more than 200 complete *Pseudomonas* genomes (Winsor et al. 2016); (2) PseudoCyc with 121 pathways and over 800 enzymatic reactions (Romero and Karp 2003); and (3) the SYSTOMONAS database for the analysis of *Pseudomonas* systems biology (Choi et al. 2007). The information is also available in commonly used databases, such as KEGG (Kanehisa et al. 2017), PubChem (Kim et al. 2016), and HMDB (Wishart et al. 2013).

In recent years, metabolomic studies of *P. aeruginosa* have also been performed. The metabolome is the set of all relatively small compounds present in the cell and released to the environment. These low molecular weight compounds (<1500 Da) play different roles as substrates, intermediates, and products of metabolism (Fiehn 2002; Pearson 2007). The information about the presence and concentration of metabolites reflects the activity of metabolic pathways in the cell. Metabolomic studies usually rely on two analytical laboratory techniques for metabolite identification and quantification: (1) mass spectroscopy coupled with chromatography (C/MS) or (2) nuclear magnetic resonance (NMR) spectroscopy.

Metabolomic studies may help solve the scientific problems unsolved by using other approaches used in system biology, such as identification of new metabolic pathways (Patti et al. 2012). These studies can provide us with data regarding virulence factors and adaptation features of a given strain to the host environment, and thereby provide a useful prognostic tool in *P. aeruginosa* infections. Due to rapid culture-independent tests, diagnosis of urgent cases and also their targeted treatment can occur quickly. These types of studies may also be used in the development of new strategies regarding the prevention and treatment of infections caused by microorganisms (Xu et al. 2014).

**Fig. 1:**
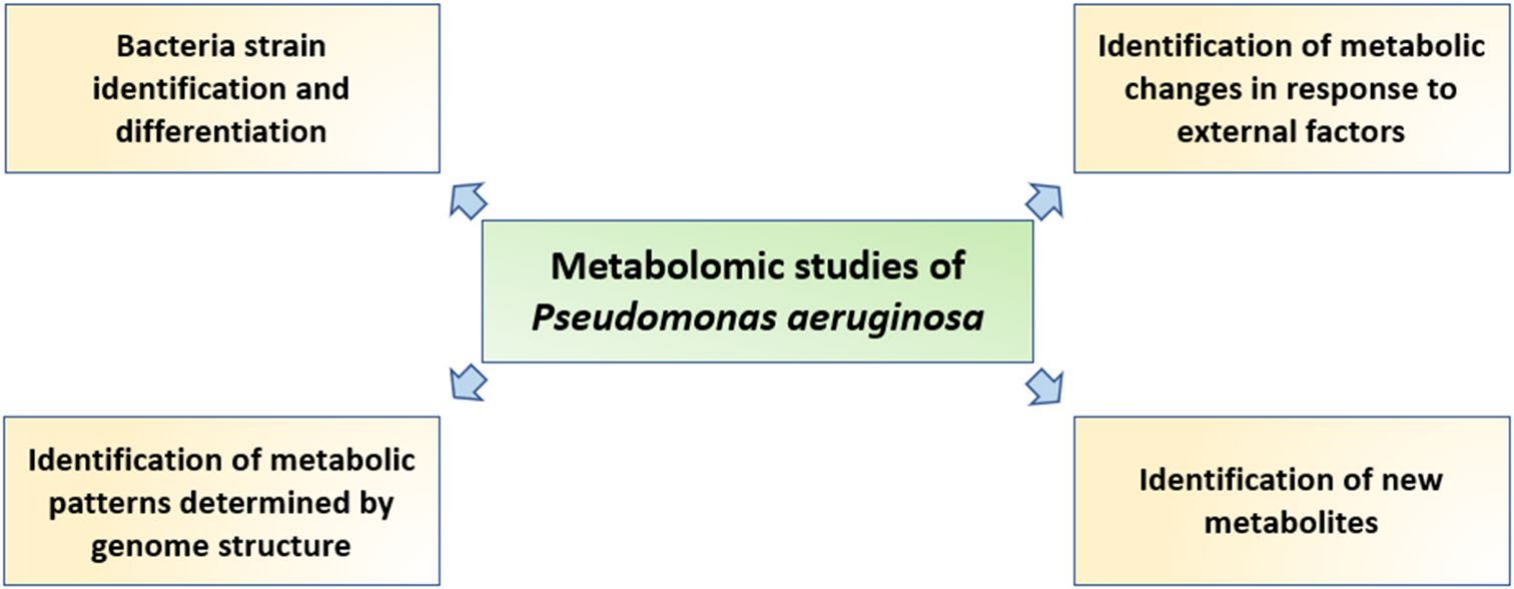
Metabolomic studies of *Pseudomonas aeruginosa*

In this article, we present a summary of the recent achievements in the field of *P. aeruginosa* metabolomics. Metabolomic studies about *P. aeruginosa* strains comparison are shown in Table 1. Studies about interactions between two species of bacteria, such as quorum sensing and co-cultures, were also conducted. The individual metabolic profile of a strain depends on internal and external factors (such as genome structure and substrate availability, respectively) (Fig. 1).

**Table 1:** Metabolomic studies comparing *Pseudomonas aeruginosa* strains

Origin of samples	Amount of samples	Type of metabolites	Measurement method	Statistic methods	Metabolites (in total)	Author, year
CF patients	179	Extracellular	^1^H NMR	Linear modelling, ‘sunburst’ plots	29	Behrends et al. (2013)
Reference strain PAO1 and CF isolate TBCF10839	2	Intra- and extracellular	GC–MS	PCA	243	Frimmersdorf et al. (2010)
CF patients	49	Extracellular	^1^H NMR	PCA, PLS, OPLS-DA	85	Kozlowska et al. (2013)
CF patient (different breeding)	1	Living cells	^1^H HRMAS NMR	Student’s t-test	24	Righi et al. (2018)
CF patients	3	Intra- and extracellular	LC–MS	PCA	221	Robroeks et al. (2010)
Clinical isolates: TBCF10839 and TBCF121838	2	Intracellular	GC–MS	Retention indices (RI)	80	Klockgether et al. (2013)
Reference strain PAO1 (different breeding)	21	Intra- and extracellular	^1^H NMR, ^1^H HRMAS NMR	PCA	–	Gjersing et al. (2007)

## A closer look at an experiment in metabolomics

Experimental design, including metabolomic analytical parts, may differ depending on the available materials, resources, and scientific questions to be answered. The identification of metabolomic changes resulting from different factors requires a distinct experimental approach. Nevertheless, the general experimental pattern is the same.

In metabolomics, as in many experiments, there are usually at least two sets of samples that are compared with one of them being the control (or reference) group. In general, metabolomic analyses cover two approaches to analyzing metabolites: (1) fingerprinting and (2) footprinting. The first contains the whole set of intracellular compounds, and the second tracks nutrient uptake and metabolite secretion (Behrends et al. 2014).

**Fig. 2:**
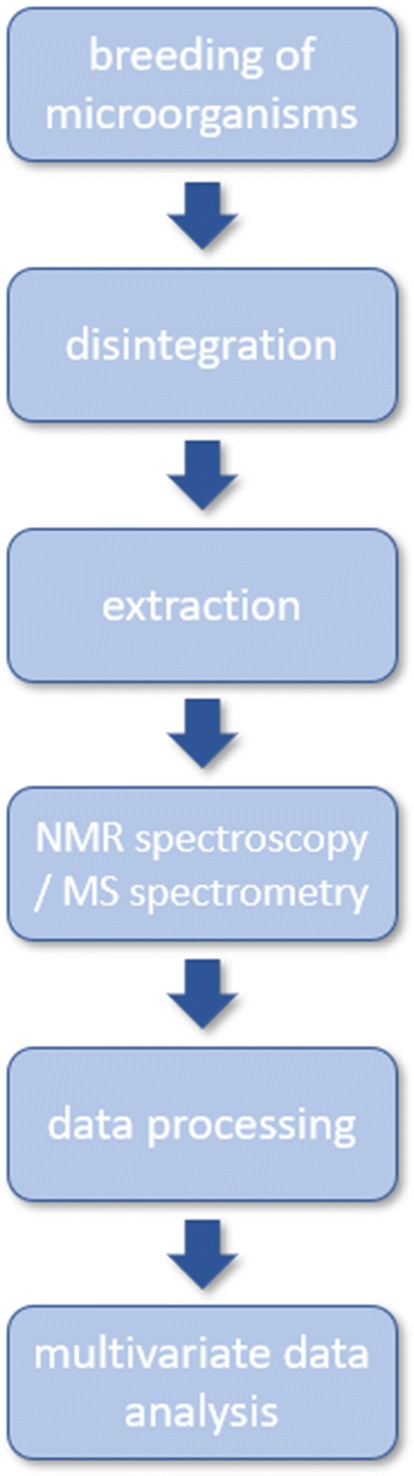
Diagram of metabolomic experiment

The typical workflow in microbiological metabolomic studies includes a few steps (Fig. 2).

Usually, in the first step, microorganisms are cultured in vitro. Appropriate disintegration (if intracellular metabolites studies are conducted) and an extraction method are then used. Metabolites from a chosen group (for instance, water-soluble) are isolated and concentrated. There are many possible approaches for metabolite sample preparation. This part of the process should be studied and depends on the purpose of the research.

In the next stage, metabolites are detected via analytical chemistry techniques. In the case of MS method, metabolites that are first separated by liquid or gas chromatography, then ionised, and detected by mass spectrometry instruments. This technique yields information about the mass to charge ratio of the analysed compounds, which could be detected by the most advanced instruments at very low femtomole to attomole detection limits. This information may be used for the identification of thousands of compounds in the sample or used as the characteristic metabolite pattern “fingerprint,” in an untargeted approach to the studied specimen. NMR spectroscopy due to application of a magnetic field allows assignment of the chemical shifts of ^1^H and ^13^C nuclei in organic compounds. This method enables identification and quantification of metabolites but at a much higher concentration than MS, which is at the mM level and strongly depends on the duration of the experiment. The second limitation is the number of compounds that can be identified, which are in the range of several dozen. However, the NMR method ensures reliable compound identification via a combination of one- and two-dimensional (1D and 2D, respectively) spectra measurements. A more detailed description of this technique may be found in the review article by Dona et al. (2016) or in dedicated handbooks. Both of these methods are complementary and mostly used in metabolomic studies.

To extract the information about metabolite type and concentration, the raw data must be further processed. The metabolic profile (list of detected metabolites with corresponding concentrations) of a single sample is still a large set of data; therefore, a comparison of samples and graphical representation of results is not easy using the conventional approach. Different statistical and chemometric methods are used to find differences and prepare data visualization. The most commonly used method in multivariate data analysis is the principal component analysis (PCA). This method is used as a starting point for further analysis. PCA is an unsupervised method, which means that the samples are underlying without any additional input data. It allows for the determination of variability and identification of outliers during all of the attempts. Additionally, it enables to determine the relationship between groups to find differentiating metabolites. PCA may be used on a raw figure without any initial metabolite identification and quantification (Gjersing et al. 2007).

The partial and orthogonal partial least squares methods (PLS and OPLS, respectively) are used to develop models, predict differences, and search for significant markers. Both of these methods are supervised methods, in which individual observations are assigned based on a specific parameter (such as membership in a given group). A more detailed description of multivariate data analysis in metabolomics can be found in an article by Worley and Powers (2013).

Information concerning a metabolome may also be stored in a database. A database dedicated to the *P. aeruginosa* metabolome was created by Huang et al. (2018). The *P. aeruginosa* metabolome database (PAMDB) provides information about > 4370 metabolites and their chemical and biological functions, more than 1250 proteins including enzymes, and almost 1000 associated pathways. Furthermore, for some compounds, NMR and MS spectra are available. The database was created based on information available in other databases and in the literature (Huang et al. 2018).

## Bacterial strain identification and differentiation

One way to conduct metabolomic experiments with microorganisms is to compare strains originating from different sources. Most studies describing strain identification use pure strains cultivated in vitro; however, a collection of a large set of metabolomic profiles is the first step in the development of methods enabling identification of strains without the need for bacteria isolation and cultivation. Such an approach would be a useful diagnostic tool because it would reduce the time between material collection and result delivery. For instance, preliminary research has proven that the analysis of volatile organic compounds (VOC) in a person’s breath might be useful for identification of respiratory tract bacterial infections; however, determination of the pathogenic strain responsible for the infection is still not possible (Maniscalco et al. 2019; Montuschi et al. 2012; Robroeks et al. 2010).

Bacterial strain identification based on the profile of volatile metabolites would be very useful in lung infection diagnoses. Nizio et al. conducted VOC profiling using gas chromatography/gas chromatography-time-of-flight mass spectroscopy (GC/GC-TOFMS) to differentiate bacteria associated with lung infections (*P. aeruginosa, Haemophilus influenzae*, *Streptococcus pneumoniae*, *Burkholderia cenocepacia*, *Stenotrophomonas maltophilia*, *and S. milleri*). Samples were analyzed in two periods: (1) short-term (between 2 and 5 days) and (2) long-term (between 48 and 50 days). Moreover, bacteria were cultured in two different growth phase conditions (stationary and logarithmic). The multivariate analysis showed that the VOC profile was sufficient for differentiation of bacteria species. However, the profiles were affected by sample storage conditions and bacterial growth phase (Nizio et al. 2016).

A similar approach for bacterial species identification was taken by Lawal et al. (2017). They investigated VOC profiles for the following bacterial species: (1) *Escherichia coli*; (2) *Klebsiella pneumoniae*; (3) *P. aeruginosa*; and (4) *Staphylococcus aureus*. These bacteria are often the cause of lung infections. To better simulate conditions in the respiratory tract, bacteria were also cultured in an artificial sputum medium. Comparison of VOC profiles was sufficient for species identification; however, profiles were considerably altered by the cultivation medium type (Lawal et al. 2017).

Moreover, in another article, Lawal et al. showed that the presence of the additional pathogen in the environment also changed the observed VOC profiles. The GC/MS method was used to identify and compare metabolites in mono and co-cultures of *P. aeruginosa* ATCC 10,145 and *Enterobacter cloacae* DSM 30,054. Among 60 VOCs identified, 24 had significantly increased and 13 decreased. Among these, under axenic cultures, bacteria-specific VOCs metabolites were identified as 2-methyl-1-propanol, 2-phenylethanol, and 3-methyl-1-butanol for *E. clonacae* while methyl 2-ethylhexanoate was characteristic for *P. aeruginosa*. However, in co-cultures, 2-methylbutyl acetate and methyl 2-methylbutyrate were found, both of which exhibited antimicrobial activity (Bail et al. 2009). In the PCA score plot, three nonoverlapping groups were observed: (1) *P. aeruginosa*; (2)* E. cloacae*; and (3) co-culture (Lawal et al. 2018).

A similar experiment conducted by Neerincx et al. also used two strains of bacteria (and co-culture): (1) *P. aeruginosa* strain ATCC 27,853 and (2) *Aspergillus fumigatus* strain AZN 8196 to compare VOCs in samples using the GC/MS method. They identified and examined 104 compounds. The PLS score plot was constructed for three-time points (16, 24, and 48 h). The analysis allowed identification of the combinations of VOCs associated with each group (*P. aeruginosa*, *A. fumigatus,* and co-culture). For each time point, specific VOC biomarker combinations were found, and individual VOCs, which were present at all-time points (for example, 8-nonen-2-one in *A. fumigatus* and 2-nonanone in co-culture), were also assigned. What is more, the location of the groups on the PLS score plot changed over time; after 48 h, the metabolic profile of the co-cultures shifted towards *P. aeruginosa* (Neerincx et al. 2016)*.* These results imply that the use of VOC profiling as a diagnostic tool may require a cultivation model that more accurately reproduces the conditions in the respiratory tract.

Palama et al. compared the bacteria responsible for urinary tract infections using the footprint approach. Using NMR, they measured the extracellular metabolites of 48 strains belonging to six species (*E. coli*, *P. aeruginosa*, *Proteus mirabilis*, *Enterococcus faecalis*, *S. aureus*, and *S. saprophyticus*). Analysis of samples collected at different growth stages identified 43 metabolites. Unsupervised multivariate data analysis showed significant discrimination between the studied samples. Furthermore, the PCA score plot showed non-overlapping groups, which originated from different microorganisms. This experiment demonstrated that metabolic profiling could be a rapid method for identifying bacterial species (Palama et al. 2016).

Kozlowska et al. recovered 15 *P. aeruginosa* isolates from sputum samples and described several culture properties, such as mucoid, pigmentation, diversity, culture pH, and others. These properties were compared with information about the subjects (age, sex, body mass index [BMI], diabetes). Analysis of the media using ^1^H NMR was performed. Statistical methods (PCA and OPLS-DA) were used to identify groups of isolates. The score plot showed four different clusters of various strains of *P. aeruginosa.* Additionally, each cluster was related to the pH of culture. Furthermore, the analysis of variance (ANOVA) test was used to find the relationship between PCA and clinical data. These experiments suggest that *P. aeruginosa* isolates have a range of growth strategies. Moreover, cluster membership was correlated with predicting patient lung function. Thus, NMR-based metabolomic profiling may be used as a prognostic tool in the diagnostics of *P. aeruginosa* infections (Kozlowska et al. 2013).

## Identification of metabolic patterns determined by genome structure

Adaptation of bacteria metabolism is crucial for microorganism survivability in different environments. In particular, pathogens change their metabolism to use available resources in a host organism in the most efficient way and to evade the host immune system (Behrends et al. 2013). Identification of critical metabolic pathways necessary for pathogen survival may open new possibilities in therapy development. It may lead to a breakthrough in the treatment of chronic infections, such as those observed in the case of cystic fibrosis patients.

Metabolic adaptation in the case of long-term infection is considered to be mainly the result of genomic changes. The comparison of closely related strains isolated from patients at different stages of infection development seems to be the best experimental approach for investigating these kinds of metabolomic alterations. Beherends et al. investigated the adaptation of *P. aeruginosa* strains to lung infections among CF patients. Exometabolomic, morphology, growth rate, and clinical data for 179 clinical isolates were analyzed. The isolates were recovered from 18 individual CF patients for 20 years. Metabolic experiments relied on NMR spectroscopy and allowed 29 metabolites to be identified. Despite the limited set of analyzed metabolites, significant changes in metabolic pathways could be identified. Strains isolated from patients suffering from long-term infection showed an improvement in amino acid uptake with a high biosynthetic cost. NMR was used to conduct exo-metabolomic analyses. This method provides a non-targeted and universal profile of all small-molecule metabolites present in cells. In total, 29 metabolites were identified, but not all of these were seen in all the samples. Nine metabolites have an association with length of infection, but most of the metabolites had no change. The exceptions were acetate, valine, serine, lysine, phenylalanine, tryptophan, trehalose, and tyrosine. Linear modelling for each metabolite against the variable ‘patient’ and ‘length of infection’ was used, and ‘sunburst’ plots for visual examination of the data were applied. This method allowed the comparison of the differences between patients and changes during infection to be followed. It also enabled the metabolomic profiling to identify the changing responses to long-term infection (Behrends et al. 2013).

A more detailed characterization of metabolome profiles was obtained using the GC/MS technique. However, the set of strains examined in this approach was relatively small. This approach was used by Klockgether et al. to compare several *P. aeruginosa* strains: (1) the reference strain (PAO1); and two strains isolated from CF patients: (2) TBCF10839 and (3) TBCF121838. GC/MS metabolomic analysis identified 80 intracellular compounds in the exponential growth phase. The concentrations of 21 compounds differed more than threefold between strains. In the case of trehalose, the level observed in strain TBCF10839 was 100 times higher than the one found in TBCF121838. The number of observed compounds in the stationary phase was similar. Moreover, this is one of the most detailed comparisons of *P. aeruginosa* strains that has ever been carried out. Apart from endo-metabolomic analysis, the experiments included several parameters: (1) genomic sequencing and comparison; (2) proteomic and transcriptome analysis; (3) exopolysaccharide phosphorylation pattern determination; and (4) phenotypic examination (Klockgether et al. 2013).

Han et al. tested polymyxin-resistant and -susceptible strains to check bacterial metabolic and lipid profile responses. In this experiment, three strains of *P. aeruginosa* (wild-type and two pmrB mutant strains) were investigated using LC/MS analysis together with DNA sequencing and genomic analysis. Various extraction methods were used for the lipidome analysis. The PCA graph showed that metabolites were grouped depending on the extraction method, and there was a difference between the wild-type strain and pmrB mutants. The metabolomic analysis allowed identification of 578 metabolites. The PCA score plot revealed the sample grouping for each strain. These studies show that mutations in the *P. aeruginosa* genome causing resistance (or lack thereof) to antibiotics are reflected in the bacterial metabolomic profile (Han et al. 2018).

Possible metabolic adaptations to oxidative stress were analyzed by Thippakorn et al. two *P. aeruginosa* strains, PAO1 and a hyperpigmented strain HP, were compared. Metabolites were identified using the GC/MS technique. The comparison of exo-metabolome revealed differences in the level of antimicrobial compounds (lower in the case of the HP strain) and antioxidant compounds (lower in the case of PAO1). Adaptation to oxidative stress was also observed at the enzyme expression level; the HP stain had a significantly higher expression of malate synthase and isocitrate lyase. These enzymes produce substrates required for the synthesis of DHN-melanin (antioxidant dye). Surprisingly, the expression of antioxidant enzymes in the HP strain was reduced in comparison to the PAO1 strain (Thippakorn et al. 2018).

## Identification of metabolic changes in response to external factors

Metabolic changes resulting from factors other than gene mutations may also play an important role in bacterial adaptation and survival. In the case of *P. aeruginosa,* several factors affecting metabolome were investigated: (1) growth medium composition; (2) growth conditions; (3) the presence of specific chemical compounds (including antibiotics); (4) other microorganisms; and (5) phage infection. Research focusing on these factors is crucial for the understanding of bacterial ecology and biochemistry. It may help to understand the mechanisms underlying phenomena, such as biofilm formation and antibiotic resistance (a considerable problem in the treatment of infections) or the mechanism of phage infection (possible alternative for conventional antibiotic therapy). Observation of metabolomic changes in response to a specific antibiotic compound may also help in the discovery of the metabolic pathways responsible for microbial resistance (Han et al. 2019).

To identify metabolic response to environmental conditions, two *P. aeruginosa* strains, PAO1 and clinical isolate TBCF10839 (responsible for CF infections), were analyzed by Frimmersdorf et al. Exo- and endo-metabolomes from different culture conditions were compared. GC/MS analysis showed the presence of at least 243 compounds. One-hundred forty-four of these compounds could be identified when compared with metabolite libraries. Sixty metabolites were found in all culture conditions, and an additional 64 were present in most of the resulting profiles. Only 65 compounds were characteristic for specific growth conditions, and the observed changes were usually dependent on the selected medium. Moreover, not all carbon sources were used, which was the case even in the stationary phase (Frimmersdorf et al. 2010).

The problem of great clinical importance is the development of antibiotic resistance. Metabolomics was used by Han et al. to understand the molecular mechanisms underlying *P. aeruginosa*-related polymyxin resistance. Polymyxins are cyclic peptides used as the last-line therapeutic option for treatment of difficult-to-treat Gram-negative pathogens. The metabolic response of two *P. aeruginosa* strains (polymyxin susceptible PAK and resistant PAK*pmr*B6) to the presence of polymyxin B (4 mg/dm^3^) was compared. The metabolites were analyzed with LC/MS techniques. Four-hundred twenty-seven hydrophobic and 871 hydrophilic metabolites were identified. Most significant changes in the metabolic profile of both strains were observed after 1 h of incubation with polymyxin B. Polymyxin induced osmotic stress in both analyzed strains as indicated by the increased level of trehalose-6-phosphate. Moreover, the PAK showed a significant decrease in lipopolysaccharide and peptidoglycan synthesis. These results may be used in the development of a new generation of polypeptide antibiotics (Han et al. 2019).

A very interesting scientific question is the influence of bacteriophage infection of the bacterial metabolome. Investigation of mechanisms associated with phage infection may result in the development of new strategies in treating bacterial infection. The influence of phage infection on the metabolome of *P. aeruginosa* was investigated by De Smet et al. (2016). They used the PAO1 reference strain and infected it during the exponential growth state with six different bacteriophages. Metabolites were detected and quantified with injection-time-of-flight MS. This approach allowed for the identification of 518 metabolites. Metabolomic profiles of infected distinguished phages relying solely on resources available in host cells and could actively modulate host biosynthesis pathways. Phage infection had a significant influence on the concentration of 24.5% of the detected metabolites. However, only 2.4% of observed alterations were common to all investigated phages. These metabolites were part of the nucleotide and sugar synthetic pathways. Amino acid metabolism is also affected by phage infection. However, the observed changes are not common and differ between individual bacteriophages. Some of the observed metabolic differences could be explained by the presence of enzymes encoded by auxiliary metabolic genes (AMG). However, the authors speculate that non-enzymatic proteins encoded by AMGs may be of equal importance. The data obtained in this project is available in the open database (https://www.biw.kuleuven.be/LoGTdb/phageBiosystems/Home.aspx).

Combined analyses of metabolome and the expression profile were carried out in the case of infection of *P. aeruginosa* PAK with PAK_P3 bacteriophage. Metabolite detection was done according to the protocol developed by De Smet et al. (2016). In this case, the pyridine metabolism was severely affected by phage infection. Moreover, the authors found that RNA-based regulation plays a central role in the PAK_P3 lifecycle since antisense transcripts are mainly produced during the early stage of infection, and viral small non-coding RNAs are expressed at the end of infection (Chevallereau et al. 2016).

In another study concerning phage infection, Zhao et al. investigated the changes in *P. aeruginosa* metabolism and gene expression after infection with the PaP1 phage. For metabolite detection, ^1^H NMR was used. The authors were able to identify and quantify 48 metabolites. In the case of 12 compounds, the observed level was significantly altered. Most changes were observed in the case of metabolites involved in energy metabolism and amino acid synthesis. Moreover, levels of NAD^+^ and betaine had considerably decreased. The authors conclude that the majority of observed changes were the result of the regulation of the host gene expression by the phage. Furthermore, they suggest that the alteration of the betaine synthesis pathway may be a potential target for therapy due to the importance of this compound for *P. aeruginosa* during infection (Zhao et al. 2017).

One of the most critical features of *P. aeruginosa* is its ability to form a biofilm. Gjersing et al. proved that the metabolome of *P. aeruginosa* planktonic cells differs from that of biofilm cells. They decided to compare the metabolomic profile of the reference strain, PAO1, with two different models of growth: (1) planktonic and (2) biofilm. For these two different types of growth, intra- and extracellular profile of the metabolites were examined. The ^1^H NMR and high resolution-magic angle spinning nuclear magnetic resonance (^1^H HRMAS NMR) methods were used. For both growth models, the recorded spectra showed different signal profiles, which showed separation between studied groups on the PCA score plot. This study demonstrates that the supernatants of biofilm and batch planktonic cultures could be readily distinguished by PCA (for both^1^H NMR and ^1^H HRMAS NMR). The results showed that the levels of metabolites in the planktonic culture were higher than in biofilm types of growth. The reason for this could have been the culture method. The planktonic culture was a standard batch fermentation without medium replacement. In biofilm culture, the medium was continuously replaced, thus metabolites produced by bacteria could not accumulate (Gjersing et al. 2007).

A fundamental phenomenon observed in bacteria is quorum sensing (QS). QS is a cell-to-cell communication mechanism, which is a biochemical mechanism that allows different bacterial groups to coordinate gene expression in a variety of environments and to also control bacterial metabolism. The functions controlled by QS are varied and depend on the needs of bacteria (Lee and Zhang 2015; Reading and Sperandio 2006). Such communication between cells plays an essential role in the creation of biofilms and infection initiation (de Kievit 2009). *P. aeruginosa* is one of the bacteria in which functioning QS plays a vital role. Reports showed that QS could be responsible for the central metabolism of this pathogen (Goo et al. 2015).

Righi et al. used ^1^HRMAS and ^1^H NMR spectroscopy to determine changes in the metabolome in live bacterial cells in response to 2-aminoacetophenone (2-AA) (Righi et al. 2018). 2-AA is considered to be a volatile quorum-sensing molecule associated with the expression of virulence factors in *P. aeruginosa* and promoting the development of chronic infection (Kesarwani et al. 2011). To understand the impact of 2-AA on the metabolome, a clinically isolated *P. aeruginosa* strain, UCBPP-PA14, was cultured with and without 2-AA. NMR analysis used whole cells without any metabolite extraction. This rapid detection method was previously optimized for UCBPP-PA14 strain and prove to be accurate for *P. aeruginosa* metabolomic analysis. Twenty-four metabolites, such as osmolytes, amino acids, and phospholipids, were identified. The combined use of 1D and 2D spectra provided complete and unambiguous metabolite identification in the samples with the conclusion that 2-AA affects the metabolic profile of cells. Changes observed in metabolome suggest that 2-AA may induce changes in the capsular polysaccharides composition and trigger cellular osmoprotectant mechanisms (Righi et al. 2013).

Chen et al. conducted an experiment in which they studied the QS inhibitor, resveratrol. The *P. aeruginosa* reference strain, PAO1, was cultured with and without resveratrol (control group). ^1^H NMR was then used to compare intracellular metabolites, which allowed 40 compounds to be identified. The PCA and PLS methods separated samples from the control cultures and resveratrol-treated cells. A reduced level of betaine and increased concentration of ethanolamine suggest the presence of oxidative stress in resveratrol-treated bacteria. Accumulation of succinate and branched-chain amino acids implies the disruption of the TCA cycle and protein synthesis (Chen et al. 2017).

Another experiment by Devenport et al. compared the influence of N-acyl homoserine lactone (AHL) on the intracellular metabolite content of two *P. aeruginosa* strains. The studies were performed using the ^1^H NMR, LC–MS, and GC–MS methods. One of the strains was the wild-type while the second was double mutant Δ *lasI rhlI*, which did not allow the production of AHL signalling compounds. MS analysis allowed fatty acids in the samples to be identified. Observation of metabolic profiles in the time intervals (from 1 to 10 h) enabled the visualization of how the metabolite concentrations changed. In the mutant’s supernatant, no AHL was detected. Furthermore, the mutant strain produced more acetate and used alanine faster than the wild-type strain. Moreover, PCA analysis clearly showed the strain grouping. The results showed that QS molecules influence fatty acid metabolism (Davenport et al. 2015).

## Identification of new metabolites

Metabolomic analysis may be a very useful tool in the identification of novel compounds produced by microorganisms. Identification of new compounds produced by microorganisms is one of the fundamental goals of present-day microbiology. Microbiologically produced substances may be significant for medicine (new drugs), industry, and environmental protection (natural biodegradable detergents) (Janek et al. 2010).

Nguyen et al. identified new lipopeptides produced by *Pseudomonas* strains using LC/MS-based metabolomic analysis. In these studies, the authors investigated 260 strains of *Pseudomonas* isolated from different locations. Massive extracellular metabolomic analysis based on the C/MS technique allowed identification of common and strain-specific compounds. For the identification of potentially novel compounds, data obtained from LC–MS/MS was processed with Global Natural Products Social Molecular Networking. Further structural analysis of strain-specific compounds based on NMR spectroscopy has led to the identification of new lipopeptides and enabled evolutionary comparisons between them. Four new compounds produced by *Pseudomonas* strains were identified, poaemides and bananamides (Nguyen et al. 2017).

## Conclusions

*Pseudomonas aeruginosa* is a very flexible and variable microorganism, which allows it to adapt to various life conditions. Chronic infection in patients with cystic fibrosis are often incurable and represent a severe problem. The adaptation of *P. aeruginosa* to the environment is a scientifically exciting problem and may be significant for therapeutic reasons. Therefore metabolomic analysis used for comparison of *P. aeruginosa* strains, causing infections among people suffering from cystic fibrosis can be very beneficial.

The presented research shows the diversity of the carried out experiments. Each of them: (1) comparing metabolome of isolates from patients suffering from cystic fibrosis with healthy people (2) characterization of compounds that make up the metabolome (3) identification of changes in metabolites during co-culture and quorum sensing; introduces a lot of new information on the functioning and dependence of this organism. The initial research showed that there are metabolome differences between strains isolated from the patients (Kozlowska et al. 2013). Observed changes included improved amino-acid uptake and reduced acetate production in strains responsible for chronic infection. However, the research also revealed great diversity between strains isolated even from one patient, thus it is hard to find any general pattern for *P. aeruginosa* adaptation strategy (Behrends et al. 2013). Moreover, it seems likely that metabolome is influenced more by the environment (medium type) than the strain genome (Frimmersdorf et al. 2010). Further research may give a better understanding of *P. aeruginosa* adaptation, however it must include a much bigger set of tested strains including environmental isolates.

Bacteriophages are considered an alternative for antibiotic therapy, especially in cases of antibiotic-resistant strain treatment. At present, the use of bacteriophages is an experimental therapy for individual cases. However, it is possible that in the future, human-designed bacteriophages will become more universal and more effective infection treatment method. The research on bacterial metabolomic changes during bacteriophage infection provides the foundations for the development of synthetic therapeutic bacteriophages.

The development of new diagnostic tools may significantly improve the therapy for *P. aeruginosa* infections. The most important information for the physician is the type of bacteria causing disease and its susceptibility to antibiotics. This type of information is critical at the beginning of therapy when a suitable and efficacious antibiotic has to be selected. The time required for data acquisition is crucial, especially in the case of life-threatening infections. Moreover, diagnostic tools are also critical in the assessment of therapy effectiveness. In the case of treatment effectiveness assessments, VOC analysis seems to be promising due to its noninvasive character and speed. However, the initial trials described in the literature were done on small groups of patients, and further tests are required.

In summary, complete identification and characterization of *P. aeruginosa* strain based on analytical multiplatform metabolic profiling is necessary. For some applications, a single method may be sufficient (treatment monitoring). The use of metabolomic analytical tools for diagnostics will be possible only after the development of an extensive database containing metabolic profiles of different *P. aeruginosa* strains. Moreover, appropriate analytical software must be used for data interpretation.

Metabolomic studies of *P. aeruginosa* has provided new interesting information about the life of this microorganism. There is still much to be done before we obtain the full scope of *P. aeruginosa* capabilities. Yet there is no doubt that the effort must be taken, since it may help us resolve the health threats associated with *P. aeruginosa* infections.
